# Supplementary material for: The genome sequence of Radford’s Flame Shoulder, Ochropleura leucogaster (Freyer, 1831)
Source: Wellcome Open Res. 2024 Dec 3;9:707. [Version 1] doi: 10.12688/wellcomeopenres.23434.1 (PMC11757926; doi:10.12688/wellcomeopenres.23434.1)
Supplement: Peer review history

# Reviewer report: Reviewer response for version 1

**DOI:** 10.21956/wellcomeopenres.25844.r114568

**Published:** 2025-01-23

**Authors:** Annabel Whibley

**Affiliations:** 1 Grapevine Improvement, Bragato Research Institute, Lincoln, New Zealand; 2 The University of Auckland, Auckland, Auckland, New Zealand

Sterling, Lees and colleagues report the genome assembly and gene annotation of Radford’s Flame Shoulder ( *Ochropleura leucogaster*), a Noctuid moth. The genome assembly is of excellent quality: all  sample properties, sequencing methods and assembly metadata are comprehensively  documented and the workflow uses current best-practice tools for assembly and evaluation. The report employs the Darwin Tree of Life data note template and this provides clearly organised data tables and figures alongside concise text describing the natural history, collection details and assembly and annotation process.  Earlier iterations of the DToL workflow did not include BOLD sample verification, and the more recent addition of this is welcomed. The public repository links are active.

Are sufficient details of methods and materials provided to allow replication by others?

Yes

Is the rationale for creating the dataset(s) clearly described?

Yes

Are the datasets clearly presented in a useable and accessible format?

Yes

Are the protocols appropriate and is the work technically sound?

Yes

Reviewer Expertise:

Genomics Bioinformatics

I confirm that I have read this submission and believe that I have an appropriate level of expertise to confirm that it is of an acceptable scientific standard.

# Reviewer report: Reviewer response for version 1

**DOI:** 10.21956/wellcomeopenres.25844.r115411

**Published:** 2024-12-30

**Authors:** Panagiotis Ioannidis

**Affiliations:** 1 Foundation for Research & Technology - Hellas, Crete, Greece

This manuscript by Sterling et al. reports the genome sequencing, assembly, and annotation of the lepidopteran insect, *Ochropleura leucogaster*.

 Being a long-read-based, the genome assembly is of high contiguity. BUSCO scores as well as other metrics clearly show that the assembly is also of high quality. Moreover, the manuscript is very well written. I particularly appreciate the quite informative figure legends, which provide enough information effectively being brief, stand-alone stories.

 Last, but most certainly not least is that the authors provide a gene set. I find that this is a significant contribution for the research community working on this group of animals, because genome annotation requires computational infrastructure, expertise and it is time-consuming.

 I only have a couple of comments:

 1) It would be very nice if the authors had run BUSCO on the gene set and provided the BUSCO scores; I believe that this is important, so that it is clear what is the level of completeness of the gene set and compare it against the completeness of the assembly. If no RNAseq data were used for gene prediction, I expect that the BUSCO scores for the gene set to be (much) lower than those from the assembly. However, it is important that any potential user knows this beforehand, since it will help him/her to draw conclusions.

 2) In Table 1, the reads counts would look much better if they were something like "590 million", or even the exact read count, with the appropriate thousands separator (e.g. 590,884,224). Writing 5.90e+08 is quite confusing in my opinion.

Are sufficient details of methods and materials provided to allow replication by others?

Yes

Is the rationale for creating the dataset(s) clearly described?

Yes

Are the datasets clearly presented in a useable and accessible format?

Yes

Are the protocols appropriate and is the work technically sound?

Yes

Reviewer Expertise:

Insect genomics, bioinformatics

I confirm that I have read this submission and believe that I have an appropriate level of expertise to confirm that it is of an acceptable scientific standard.
